# The choroid plexus and its role in the pathogenesis of neurological infections

**DOI:** 10.1186/s12987-022-00372-6

**Published:** 2022-09-10

**Authors:** Derick Thompson, Catherine A. Brissette, John A. Watt

**Affiliations:** Department of Biomedical Sciences, School of Medicine and Health Sciences, University of North Dakota, Grand Forks, ND USA

**Keywords:** Choroid plexus, Infection, Blood–CSF barrier, Immunity, Neuroinflammation, Hydrocephalus, ZIKA virus, SARS-CoV-2, Borrelia burgdorferi, John Cunningham virus

## Abstract

The choroid plexus is situated at an anatomically and functionally important interface within the ventricles of the brain, forming the blood-cerebrospinal fluid barrier that separates the periphery from the central nervous system. In contrast to the blood–brain barrier, the choroid plexus and its epithelial barrier have received considerably less attention. As the main producer of cerebrospinal fluid, the secretory functions of the epithelial cells aid in the maintenance of CNS homeostasis and are capable of relaying inflammatory signals to the brain. The choroid plexus acts as an immunological niche where several types of peripheral immune cells can be found within the stroma including dendritic cells, macrophages, and T cells. Including the epithelia cells, these cells perform immunosurveillance, detecting pathogens and changes in the cytokine milieu. As such, their activation leads to the release of homing molecules to induce chemotaxis of circulating immune cells, driving an immune response at the choroid plexus. Research into the barrier properties have shown how inflammation can alter the structural junctions and promote increased bidirectional transmigration of cells and pathogens. The goal of this review is to highlight our foundational knowledge of the choroid plexus and discuss how recent research has shifted our understanding towards viewing the choroid plexus as a highly dynamic and important contributor to the pathogenesis of neurological infections. With the emergence of several high-profile diseases, including ZIKA and SARS-CoV-2, this review provides a pertinent update on the cellular response of the choroid plexus to these diseases. Historically, pharmacological interventions of CNS disorders have proven difficult to develop, however, a greater focus on the role of the choroid plexus in driving these disorders would provide for novel targets and routes for therapeutics.

## Introduction

The choroid plexus (CP) is a highly vascularized complex found within each of the four ventricles of the brain. It is comprised of a monolayer of polarized secretory epithelial cells whose primary role is the production and secretion of 60–75% of total cerebrospinal fluid (CSF) [1]. The anatomy of the CP reflects this role. The epithelial surface, which is a continuation of the ependyma that lines the ventricles, becomes highly folded and forms villi throughout its structure. On their apical side, the cells present a brush-border of microvilli that greatly increases surface area to enable high rates of exchange of water and solutes. At its core lies a stroma of connective tissue and highly fenestrated capillaries [2, 3] that permit the diffusion of fluid and small molecules into the stroma. Together, this allows for the rapid production of CSF in which, for humans, the total volume (150 ml) of CSF is circulated and replaced approximately three to four times per day [4]. As there are no tight junctions between the endothelia of the CP, the epithelium functions similarly to the blood–brain barrier in regulating the passage of peripheral substances into the central nervous system (CNS).

The blood-cerebrospinal fluid barrier (BCSFB) is formed by the epithelial layer of the CP through the expression of junctional complexes forming a tight barrier segregating the highly vascularized stroma and the CSF of the ventricles. Like the blood–brain barrier (BBB), the BCSFB aids in the separation of the peripheral and central systems, maintaining a highly regulated environment. Although the ependymal cells that line the ventricles express some junctional components, they are loosely connected by desmosomes and any tight junctions tend to be discontinuous, representing a leaky CSF-brain interface [5]. Thus, any pathogens that cross this barrier into the CSF can greatly influence the homeostatic state and have direct access to the brain parenchyma. Moreover, disruption of the BCSFB can lead to indirect damage to the nervous system through inflammatory mechanisms and autoimmunity within the CSF [6]. As such, the choroid plexus houses many types of immune cells primarily within the stroma, including macrophages, dendritic cells, and T cells, all of which contribute to local immunosurveillance. Additionally, the choroid plexus has been shown to provide a point of entry into the CSF for peripheral immune cells. Historically, considerable attention has focused on the BBB for its role in the pathogenesis of infection into the CNS, in part due to its large interface with the brain [7, 8]. However, there has also been substantial evidence showing that the BCSFB provides a site of entry for many pathogens and may play a major role in modulating the immune response of the CNS. Suggesting that the BCSFB may not just be a potential site of entry but a preferential site for some infections.

In recent years, our understanding of the CP and its role in CNS immunity has been greatly improved. The wide-spread use of barrier model systems has provided insight into the bidirectional crosstalk between the peripheral and central systems at the CP interface and, with new understandings in CSF production and reabsorption, novel mechanisms that underlie disorders such as post-infectious hydrocephalus (PIH) are being uncovered. Novel model systems such as the choroid plexus organoid system allows for the manipulation and study of CSF production in conjunction to barrier functions [9]. This review covers experiments that utilize diverse model systems that have unique benefits and limitations [10–13]. Furthermore, with the use of single-cell sequencing, detailed mapping of the CP across brain ventricles and developmental ages has been performed, providing insight on the cellular makeup and transcriptional shifts throughout maturation [14]. The goal of this review is to highlight anatomical and functional aspects of the choroid plexus that are relevant to its role in the pathogenesis of neurological infections, and to bring attention to future research and potential therapeutics.

## Anatomy and function of the choroid plexus

### CSF production

The choroid plexus epithelial cells are responsible for the primary production of CSF which occurs through the cotransportation of water and ions [15]. The production of CSF is a complex process that requires the active and passive transport of solutes and water which rely on changes in an osmotic pressure gradient. As such, there is still debate on the mechanisms that underlie CSF production by the CP, and in fact opposing hypotheses suggest that the CP may not be the main producer of CSF [16]. However, the current generally accepted process of CSF production and secretion is thought to occur in two discrete stages. First, the fenestrated capillaries within the CP allow for an ultrafiltrate of plasma to passively permeate into the basal lamina. The fenestrae permit the free flow of water, ions, and small molecules dependent upon the pressure gradient between the blood and interstitial fluid. Secondly, the fluid then must undergo active transport across the epithelium barrier (1C) [17].

**Fig. 1:**
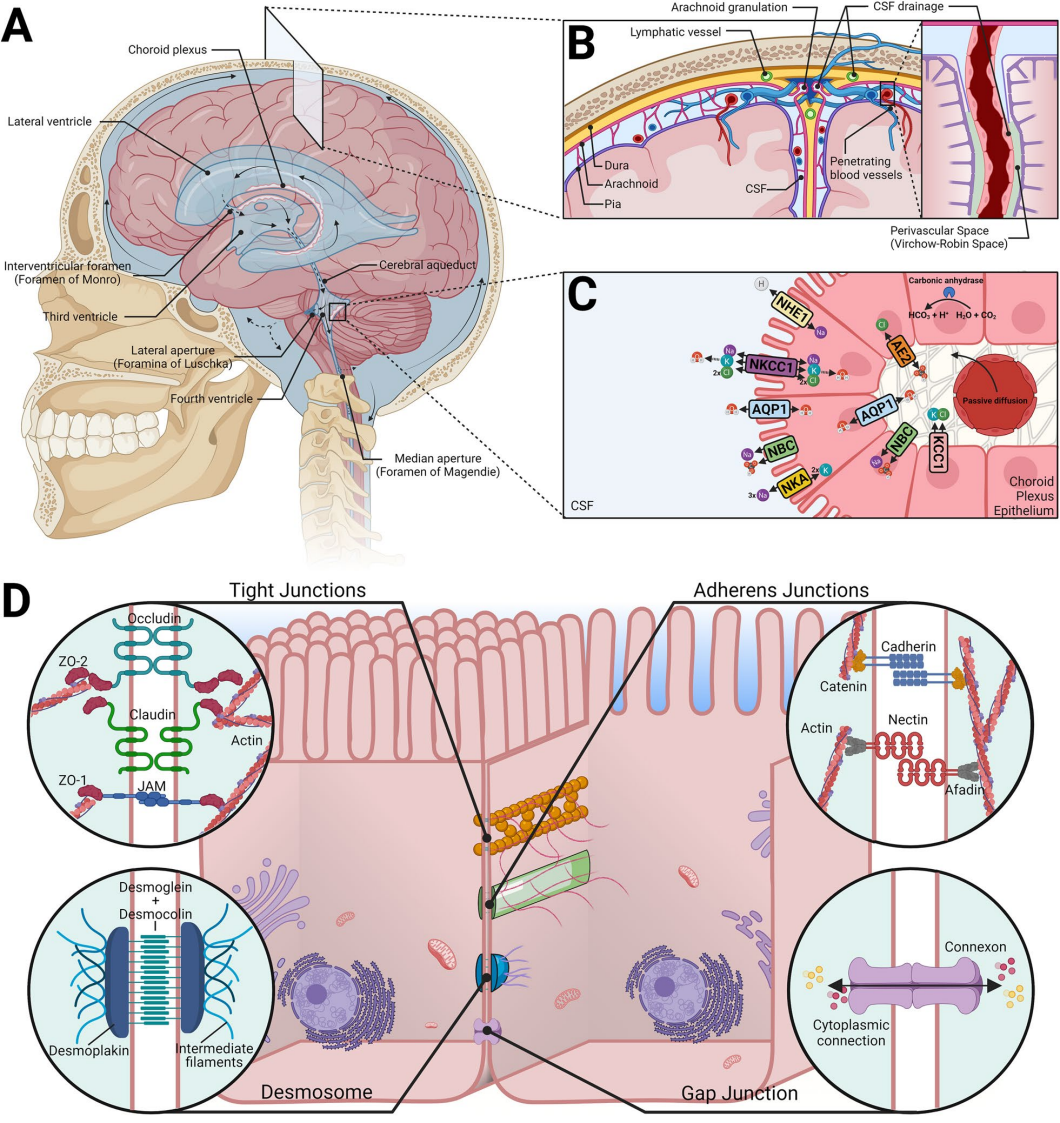
Overview of CSF production and junctional properties of the blood-CSF barrier. **A** A diagram indicating the bulk flow of CSF following its production from the CP. The CSF flows in a unilateral direction through the ventricular system, and multidirectionally throughout the subarachnoid space. **B** A coronal section showing the drainage of CSF. Three distinct sites of CSF drainage are highlighted—through dural lymphatics, arachnoid granulations, and through perivascular space (inset) where it enters the brain parenchyma and exits via perivenous routes. **C** A molecular view of CSF production and several important transporters. Passive diffusion of water and solutes occur through the fenestrae of the blood vessels. Differential transport of ions across the membrane creates an electrochemical gradient that drives water transport via AQP1. Direct coupling of water with transporters occurs, NKCC1. **D** An overview of the polarized junctional proteins found in the CP epithelium – tight junctions, adherens junctions, desmosomes, and gap junctions are present, along with their scaffolding proteins. Created with BioRender.com

The secretion of fluid by the choroid plexus epithelial cells is dependent on the unidirectional transport of specific ions because of polarized expression of transporters. This leads to an osmotic gradient that causes water to move from the stroma, across the epithelial barrier in either a paracellular or transcellular fashion and enter the ventricular lumen. The primary determinants of this exchange are Na^+^, K^+^, Cl^−^, and HCO_3_^−^ [18]. Following the diffusion of water and CO_2_ into the epithelial cells from the interstitial fluid, cytoplasmic carbonic anhydrases catalyze the production of HCO_3_^−^ and H^+^ [19]. The accumulation of intracellular HCO_3_^−^ and H^+^ is then exchanged for Na^+^ and Cl^−^ through the SLC4 (anion exchange proteins [AEs] and sodium bicarbonate cotransporters [NBCs]) and SLC9 (sodium-hydrogen exchanger [NHEs]) family of transporters located on the basolateral membrane [20–22]. Na^+^-K^+^ ATPase (NKA) pumps play a vital role in the secretion of Na^+^ into the CSF, and conversely, the uptake of K^+^ into the epithelial cells. These ATPases are localized at the apical brush border which exchange three Na^+^ ions for two K^+^ ions at the expense of one ATP [23]. The importance of these have been observed through inhibitor studies that reduce CSF production by up to 80% [24]. Furthermore, a family of electroneutral cotransporters (SLC12 family—NKCCs) are responsible for the movement of Cl^−^ with Na^+^ and/or K^+^ across the cell membrane. The movement of Cl^−^ via the SLC12 family members is driven by changes in the chemical gradients of Na^+^ and K^+^ [18]. Ultimately, this series of passive diffusion and active ion exchange across the polarized epithelial membrane creates an osmotic gradient that drives the secretion of water into the CSF that is facilitated by aquaporins [15]. Figure 1C highlights this process.

Recent studies have shown that knockout of AQP1 results in decline of CSF production by only 20% and that the observed osmolarity across the membrane (5–10 mOsm) does not reach the calculated amount (250 mOsm) required for the observed rate of CSF production [4, 25–27]. In fact, the choroid plexus has been shown to be able to transport water against the direction of the osmolarity gradient through cotransporter channels [26, 28, 29]. Therefore, it is suggested that one of the main contributors for water secretion and thus CSF production occurs through the direct coupling of water to the Na^+^/K^+^/2Cl^−^ and K^+^/Cl^−^ cotransporters (NKCC1 and KCC respectively) [27, 29, 30].

Following secretion, CSF generally flows directionally through the ventricular system in a pulsatile fashion that corresponds to the CP systolic pulse wave of the arteries and aided by the movement of ependymal cilia [1, 31]. CSF produced within the lateral ventricles will pass through the interventricular foramina and into the third ventricle. From here it then passes through the cerebral aqueduct and enters the fourth ventricle. The CSF then leaves the ventricular cavities and flows multidirectionally throughout the subarachnoid space and spinal canal through the central canal of the spinal cord, the foramen of Magendie, and the foramina of Luschka [1, 32]. Classically, CSF drainage into the vascular system was thought to occur through the absorption of CSF by arachnoid granulations that protrude into the dural venous sinuses. However, the predominance of this pathway has come into question with new routes being discovered [33]. For example, meningeal lymphatics play an important role in the transport and drainage of CSF passing through the cribriform plate into the nasal lymphatics and cervical lymph nodes [34–38]. Recent research has emphasized the significance of this lymphatic drainage system due to its potential role in modulating the pathophysiology of many neurological diseases and disorders, as well as regulating neuroinflammation through the transport of CSF immune cells and antigens into lymph nodes [39, 40]. This lymphatic drainage allows for constant immune surveillance through the flow of CSF antigens and immune cells. In the healthy CNS, this allows for self-tolerance of brain-derived antigens; however, during neuroinflammation, this allows for antigen presenting cells and circulating T cells to relay inflammatory information to the periphery during tissue damage or innate immunity activation [40, 41]. This migration of immune cells to the draining lymphatics occurs in a CCR7-dependent manner, that is enhanced during immune activation [40–42]. In addition, the expression of CCL19/21 chemotaxis is regulated by proximal lymphatic vessels. Importantly, during experimental autoimmune encephalomyelitis, the ablation of meningeal lymphatics reduces pathology and inflammation [40–42]. The role of the meningeal lymphatics system in modulating and communicating CNS inflammatory and immunity to the periphery has received considerable attention in regards to autoimmunity. A common theme amongst CNS infections is the invasion of peripheral immune cells, specifically T cells into the CSF, that may be activated through initial drainage of antigens or antigen presenting cells through the meningeal lymphatics. The importance of this lymphatic system during CNS infection should not be understated and requires greater research to determine potential avenues in modulating CNS pathology.

In addition to the lymphatics system, CSF and solutes have been observed to also flow through the perivascular space (Virchow–Robin space) of penetrating blood vessels and entering the brain parenchyma through aquaporin-4 on astrocytic endfeet [43]. Along with the interstitial fluid, the CSF is then drained via perivenous pathways [43]. Figure 1A and B illustrate the bulk flow and drainage of CSF.

### Barrier properties and junctional proteins

The choroid plexus epithelia also form the blood–cerebrospinal fluid barrier. Like the structural organization of transporters, the junctional proteins that comprise the BCSFB are highly polarized. As indicated in Fig. 1D, these proteins are spatially oriented from the apical to basolateral side. [44–47]. Together, these protein complexes allow for a highly regulated selectively permeable barrier to paracellular diffusion and cellular movement. Importantly, the barrier can maintain the semi-immune privileged state of the CNS by limiting the movement of peripheral immune cells and pathogens across the membrane. Thus, an uncompromised barrier is integral to maintaining a healthy homeostatic environment and modulating CNS immunity.

The primary functions of tight junctions are to act as a gate-like barrier between adjacent cells that regulates the paracellular movement of water, ions, and macromolecules, and to establish and maintain cellular polarity by preventing the redistribution of lipids and membrane bound proteins between the apical and basal surfaces [48]. The junctions are formed by transmembrane proteins that include occludins, claudins, and junctional adhesion molecules (JAMs) and can be found throughout the choroid plexus epithelium [45, 49–51]. The protein’s extracellular domains from adjacent cells bind directly to each other and seal the paracellular pathway, allowing for the regulation of ions and solutes. However, the permeability of these tight junctions, including JAMs, can change depending on their conformation and can be disassociated based on their phosphorylated state [52, 53].

Gap junctions provide minimal adhesive properties and do not form a tight seal between adjacent cells, but instead they form intercellular channels that directly connect the cytoplasm of cells [54]. These channels, formed by proteins called connexins, provide a route of communication to coordinate and maintain a homeostatic environment that are often required in many blood-tissue barriers, including the blood–brain barrier [55]. This form of communication allows for dynamic changes to occur in response to cellular stress, inflammation, or infection [55]. Their roles within the BCSFB have been largely ignored, which represents a major gap in our understanding of the barrier properties of the choroid plexus and how pathogens may compromise these complexes, especially during fetal development.

### Receptors and adhesion molecules

**Fig. 2:**
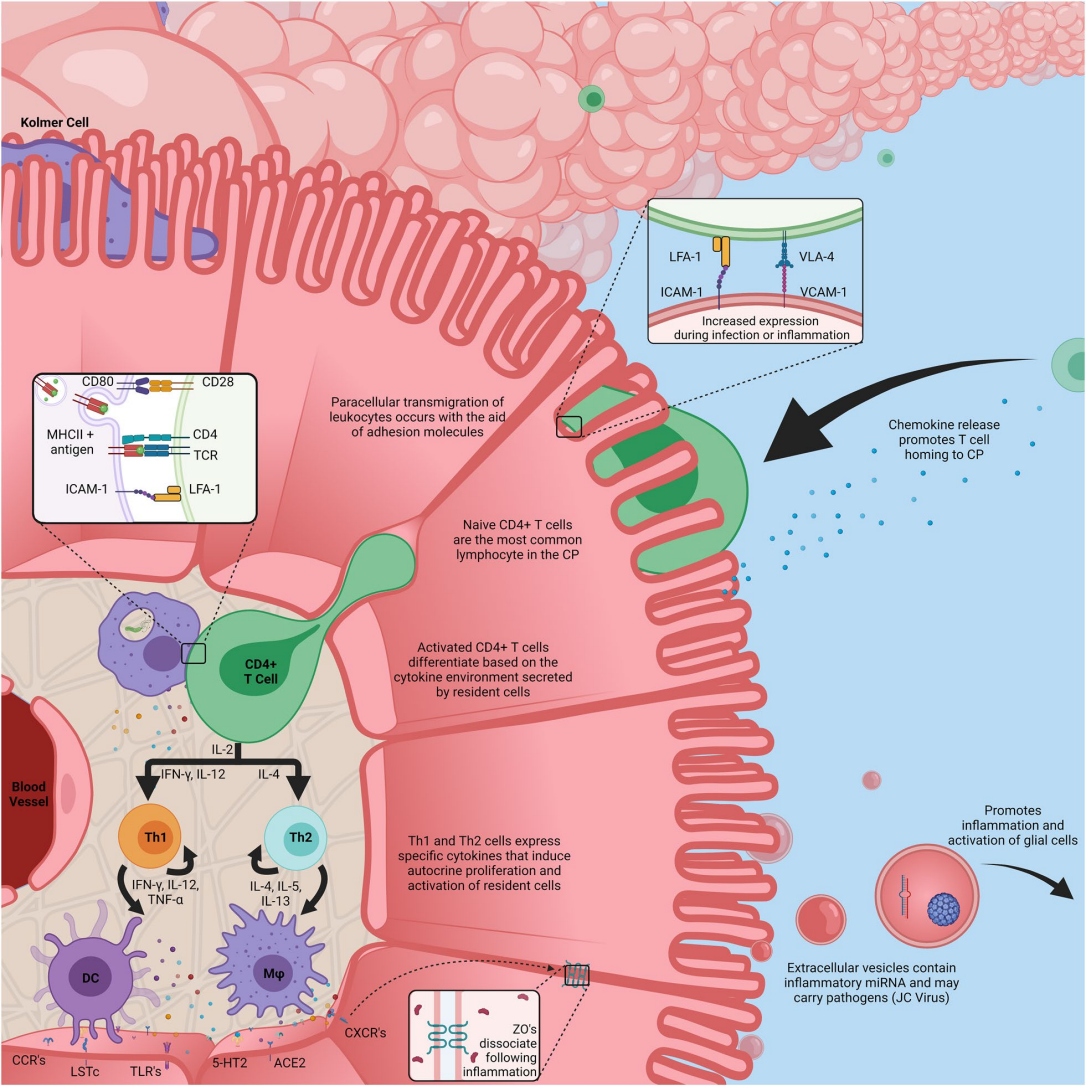
An illustration of the choroid plexus, receptors, and resident immune cells. Three distinct immune cells can be found within the CP–dendritic cells, macrophages, and CD4 + T cells. Kolmer cells can be found adhering to the apical surface of the epithelium. CD4 + T cells are seen to constantly surveil both the CP and CSF, and regular traverse the barrier bidirectionally through the aid of adhesion molecules. The CP epithelium express many receptors and can be activated by resident immune cells, pathogens, or circulating cytokines from the periphery. Dendritic cells and macrophages phagocytize pathogens and present antigens to naïve T-cells. This allows for differentiation of CD4 + T cells into Th1 or Th2 dependent upon the current cytokine environment. The epithelial cells can release chemokines in the stroma and CSF. This can alter the CSF composition and promotes homing of peripheral immune cells to and from the CSF. The epithelial cells can also release extracellular vesicles into the CSF that contain inflammatory miRNA and potentially pathogens. Created with BioRender.com

The choroid plexus is a highly vascularized structure, and in combination with the fenestrae of the blood vessels, provides an interface between the peripheral circulation and the CNS. Thus, like many epithelial barriers, the cells of the BCSFB contain many types of immune receptors and adhesive molecules to surveil and sample the microenvironment of the stroma (Fig. 2). A significant feature of the innate immune response is pattern recognition receptors (PRRs) to identify pathogen-associated molecular patterns (PAMPS). These receptors induce an innate immune response and give rise to inflammatory pathways through the release of cytokines which can lead to greater BCSFB permeability. Inappropriate stimulation of these receptors by infection and subsequent inflammation at the choroid plexus or in adjacent tissue can lead to devastating outcomes such as meningitis, hydrocephalus, hemorrhage, and death [56–59]. Thus, many studies have focused on the impact of peripheral infection and systemic inflammation on BCSFB integrity and the transmigration of both pathogens and immune cells.

The most studied PAMP receptor within the choroid plexus are the toll-like receptors (TLRs). Several studies have sought to determine the expression pattern of TLRs in the CP in mice, rats, ewes, and in vitro human systems in different physiological states [60–67]. There has been a total of 13 TLRs identified so far and it is important to note that the presence of TLRs vary between species, with TLR1-10 being found in humans, and all except TLR10 being found in mice. Furthermore, tissue localization of TLRs varies substantially and as such the expression of TLRs within specific model systems of the choroid plexus, such as human choroid plexus papilloma cells, should be carefully considered. Nevertheless, considerable research has shown that TLRs of the CP epithelial cells play a fundamental role in CNS immunity and inflammation. Lipopolysaccharide (LPS) and Pam_3_CSK_4_ (PAM), ligands for TLR-4 and TLR-1/2 respectively, are commonly used to study the effects of systemic inflammation on barrier function. Challenging with TLR agonists in animals and in in vitro models commonly show an increase in the expression of inflammatory genes and a downregulation of junctional proteins, including claudins and cadherins [64, 68, 69]. Many of the inflammatory genes promote the activation of resident immune cells and act as chemoattractants for circulating immune cells, suggesting that stimulation of TLRs leads to cytoskeleton restructuring and loss of junctional complexes, and promotes peripheral immune cell migration to the choroid plexus [64, 67–69]. In fact, the activation of the TLR1/2 complex by PAM has been shown to induce a significant increase in both cytokine levels and leukocyte count within the CSF [68, 70]. These studies have provided mechanistic insight into disease pathogenesis of the CNS as their effects tend to reflect those observed in many infectious models. Other types of PAMP receptors that have been shown to be important in mediating immunity, including NOD-like receptors, C-type lectin receptors, and RIG-I-like receptors have received considerably less attention within the CP, but may play important roles in the exacerbation of CNS inflammation as many have been shown to be associated with TLR-signaling [71–74]. In fact, a study by our laboratory showed an increase in the RIG-I-like receptor and components within its signaling pathway when human CP epithelial cells were stimulated by the Lyme disease bacterium, suggesting a potential role for this receptor in the CP immune response [73].

In addition to PRRs, CP epithelial cells contain a myriad of cytokine receptors that can respond to ligands in circulation or from the CP itself if challenged. TNFα, a pro-inflammatory cytokine that is produced in periphery tissue as well as by the CP epithelia, can cause deleterious effects on tight junctions, an increase in cell-adhesion molecules utilized by immune cells (ICAM and VCAM), and an increase in matrix metalloproteases (MMPs) [75–77]. Collectively, MMPs are enzymes that are capable of cleaving and remodeling many components of the extracellular matrix. The dysregulation of MMPs is associated with many neuroinflammatory disorders and in fact, some MMPs, such as MMP-2 and MMP-9, are indicative of specific infectious diseases as they show a consistent elevation in CSF concentrations [78–81]. Furthermore, with the use of an MMP inhibitor, Zeni et al. showed that alterations of the BCSFB induced by TNFα were in part dependent on MMPs [82]. More recent studies corroborate and expand upon the role of MMPs showing that barrier integrity is compromised via the NF-κB pathway and subsequently led to the degradation of claudins [75, 83]. Several cytokines that are found in peripheral circulation following infection, including IL-1β, IL-6, and IFNs have been shown to stimulate similar inflammatory profiles that, in turn, may lead to compromised barrier integrity [84–86]. As the choroid plexus itself can produce these inflammatory signals, it can be suggested that a positive feed-back loop, if not properly regulated, would lead to a more deleterious outcome in disease pathology.

While it is well-known that CP epithelial cells release cytokines into the CSF following PRR activation or other stressors and can influence brain inflammation, a novel mechanism of blood–brain communication at the CP was discovered [87]. CP epithelial cells can release extracellular vesicles into the CSF, and their release can be increased through systemic peripheral inflammation (LPS injection). These vesicles contained miRNAs that are then taken up by astrocytes and microglia, inducing an inflammatory program [87]. This pathway has the potential to shuttle pathogens between the periphery and brain parenchyma, a subject that has seen very limited research—recently the John Cunningham virus was shown to have the ability to utilize this pathway in infecting glial cells, which typically lack the necessary viral receptors [88].

### Resident immune cells

Under physiological conditions, there are typically 3 types of immune cells that can be commonly found within the CP stroma or adhered to the apical side of the CP epithelia. These are macrophages (Epiplexus/Kolmer cells), dendritic cells, and T-cells (Fig. 2) [89–94]. Two subtypes of macrophages can be found to be in contact with the CP–stromal CP macrophages and Kolmer cells that adhere to the apical side of the epithelial barrier and ventricular wall. Both cells share similar functional characteristics in that they are phagocytically active, and are antigen presenting cells, aiding in the activation of T-cells [90, 95]. Kolmer first reported macrophage-like cells on the surface of the CP epithelium of amphibians in 1921, and subsequent studies further reinforced these findings in other vertebrates, including humans [90]. It wasn’t until the 1970’s in which electron microscopy aided in the functional and morphological characterization of these cells, with later studies identifying phagocytic activity [96, 97]. Immediately after their discovery, the ontology of these cells came into question. The prevailing theory was that of myeloid origin in which circulating monocytes infiltrated the CP stroma, differentiated into tissue macrophages, and subsequently migrated across the epithelial barrier to become Kolmer cells [90]. As such, and due to the lack of differentiating markers and characteristics, stromal macrophages and Kolmer cells have commonly been evaluated together in many CP studies. However, a recent study utilizing single-cell sequencing determined distinct profiles between Kolmer and stromal cells. It was shown that while both populations are originally yolk sac-derived, stromal macrophages are gradually replaced by circulating monocytes while Kolmer cells were capable of repopulation, independent of bone marrow progenitors [98, 99]. Interestingly, while Kolmer cells showed distinct clustering and shared many characteristics with stromal macrophages, these cells also expressed several signature genes typically found in microglia, including and *Sparc* and may suggest that they are instead a subset of microglia [98, 100, 101]. However, further characterization is needed in order to fully delineate the lineage of these macrophages throughout development. A recent review by Cui and Xu et al. detail the immunological role and heterogeneity of macrophages in the CP [102].

Dendritic cells (DCs) are functionally similar to macrophages in that they may act as antigen presenting cells and are capable of phagocytosis. As DCs are some of the first cells to encounter a pathogen when invading a host, and are present at the interface of the BCSFB, they play an integral role in bridging the innate and adaptive immune response. Following activation, dendritic cells are able to secrete a range of inflammatory cytokines including TNF-α, IL-1, IL-6 which stimulate and promote the release of chemotactic chemokines by the CP epithelium leading to T-cell activation and differentiation [103]. Through these mechanisms, DCs initiate an innate immune response, leading to CP inflammation and the further migration and activation of peripheral immune cells. This creates an environment that is conducive to the transmigration of both immune cells and pathogens across the BCSFB, and gives rise to an increase of cytokines and immune cells within the CSF, a hallmark of many neurological infections [104, 105].

Under normal physiological state, the choroid plexus has been found to be populated with T-cells, with the majority being effector memory CD4+T Cells, and a smaller population of CD8+T cells [106]. During infection or inflammation, MHCII + resident immune cells have been shown to closely associate with T cells, suggesting antigen presentation and activation prior to T-cell invasion of the CSF [93, 107]. Additionally, the CP epithelium plays an important role in the activation and transmigration of T-cells across its border. Aside from secreted chemokines that activate and attract immune cells, the epithelial cells constitutively express cellular adhesion molecules, including VCAM-1 and ICAM-1, both of which are important for adhesion of T-cells and required for transmigration into the CSF [108, 109]. Furthermore, polar expression of these adhesion molecules can be observed on the apical side of the epithelial cells and expression is found to increase during inflammation and infection [110, 111].

The presence of B cells within the CP during infection is poorly understood. However, in many forms of autoimmunity (multiple sclerosis and lupus), deposits of immunoglobulins can be observed, as well as the accumulation of B cell subsets within the CP and CSF [112–114]. There is evidence showing that AIDS patients and a patient with subacute bacterial endocarditis form immune complex deposition at the CP [115, 116]. In 75% of patients with AIDS, immunoglobulin deposits were observed, however, as circulating immune complexes are common in AIDS patients and there was a lack of CP pathology, it is suggested that their origin stems from the bloodstream, as opposed to B cell infiltration [115]. In a rodent model of malaria, circulating immune complexes and depositions were also found within the CP [117]. These observations make it clear that the antibody immune response impacts the CP in many circumstances in both autoimmunity and infection and may in fact be a common occurrence that is poorly studied. A study of MS patients showed preferential localization of antibodies at CP epithelium [118]. While antibodies can be observed circulating throughout the CSF during disease, it is not clear how the choroid plexus plays a role in their transport. Some studies suggest that the localization of antibodies at the CP epithelium is due to the efflux (CSF to blood) of these immunoglobulins via the FcRn-dependent IgG transcytosis pathway [118–121]. Our current understanding suggests that the CP may act as a trap, pooling immunoglobulins within the stromal matrix, and protecting the brain from immune-mediated damage [118].

## The cellular response of the choroid plexus to specific pathogens—an update

**Fig. 3:**
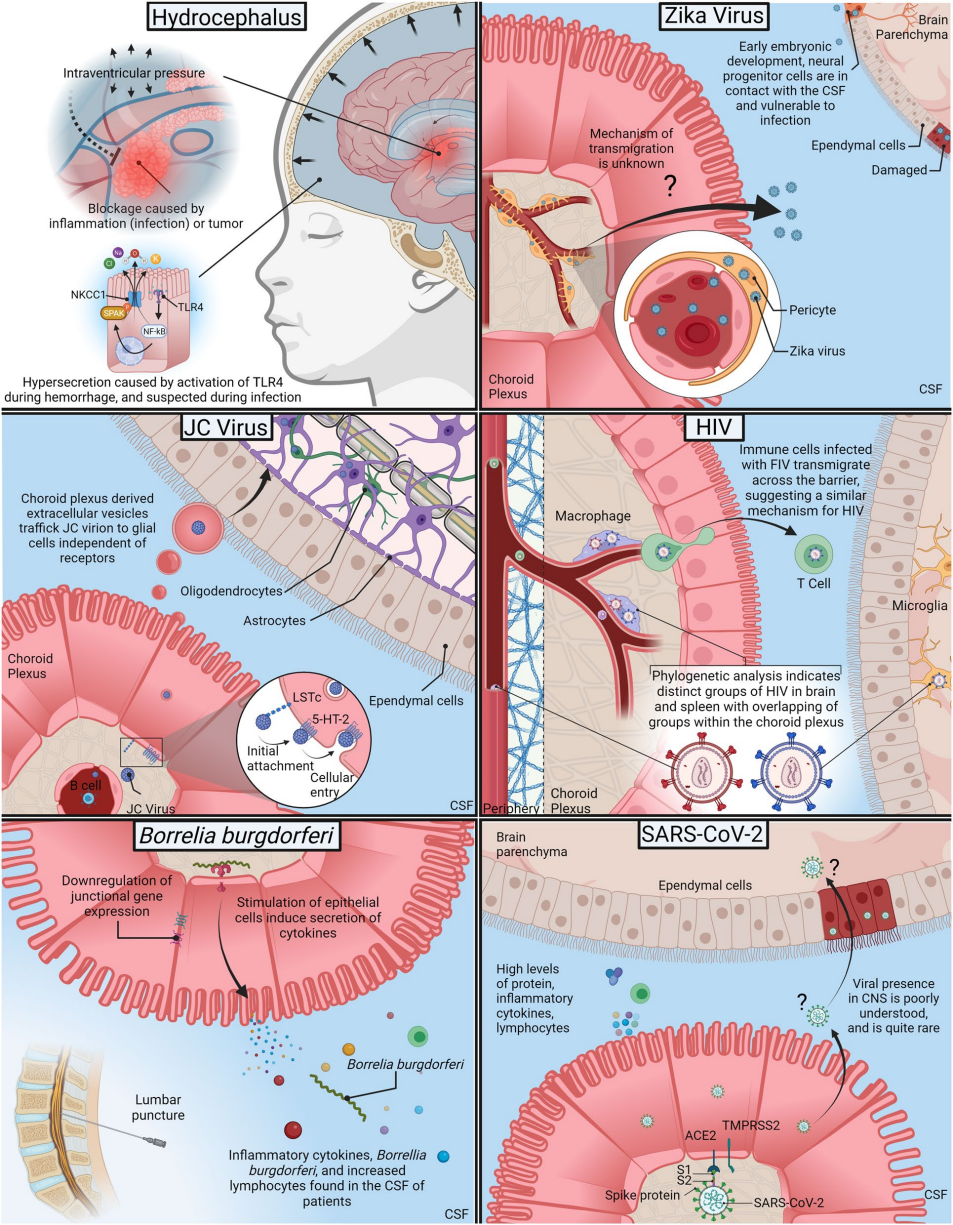
Summary of pathogens and their interactions with the choroid plexus. (Hydrocephalus) Inflammation can cause obstruction of CSF flow from the lateral ventricles leading to increased intraventricular pressure. Ventricular hemorrhage and infection can induce inflammation dependent hypersecretion of CSF, promoting hydrocephalus. The hypersecretion occurs via TLR4-NF-κB and the NKCC1 transporter. (ZIKA) The ZIKA virus is shown to preferentially infect pericytes within the CP. This infection precedes CSF and brain invasion—the mechanism of transmigration unknown. Depending on the developmental stage, neural progenitor cells may be exposed to the CSF and thus susceptible to infection. (JC Virus) During the lytic phase, JC virus may disseminate hematogenously to the CP or possibly by B cells. The CP epithelial cells express viral receptors and are susceptible to infection. Extracellular vesicles secreted by the CP into the CSF can contain JC virion and transport these to glia in the brain. (HIV) HIV can be found within the CP, which may act as a reservoir for viral replication. Phylogenetic analysis indicates distinct clustering of HIV in the brain and spleen, whereas the CP contains virus from both clusters. This suggests there is unique selective pressure within the CP towards CNS tropism of HIV. FIV is capable of transmigrating across the BCSFB. (*Borrelia burgdorferi*) CSF findings indicate increased inflammatory cytokines, lymphocytic pleocytosis, and *Bb*. Infection of CP epithelial cells with *Bb* induce an increase in secretion of inflammatory and chemotactic cytokines, as well as the downregulation of junctional proteins. (SARS-CoV-2) Early findings suggest that viral presence in CNS is rare but neurological complications more common, characterized by increased cytokines and lymphocytes in the CSF. The CP expresses binding receptors for viral fusion. The CP may provide a route of entry for SARS-CoV-2 in rare circumstances, or more likely, relay inflammatory signaling to the CNS. Created with BioRender.com

The goal of this review is to bring attention to recent major developments in our understanding of the CP immunity during infection. In the past several years, thorough reviews have been published describing the impact of specific pathogens and their molecular pathways for migration (reviewed here: [6, 92, 122–124]). Since then, there has been an exponential focus on the CP, in part due to several high impact diseases that have affected the world, including Zika and SARS-CoV-2. Notable advancements in our knowledge of CP function during infection has prompted the need for an update. Figure 3 highlights our current understanding of these diseases that are being discussed.

### Hydrocephalus

Hydrocephalus is a condition in which abnormal amounts of CSF accumulates within the CNS, typically resulting in increased intracranial pressure, macrocephaly, cognitive dysfunction, and death, if not properly diagnosed and treated. Although hydrocephalus has a wide range of etiologies, including congenital malformations, trauma, or tumor formation, research has shown that the most common cause world-wide is induced by infection, termed post-infectious hydrocephalus (PIH) [125, 126]. Furthermore, recent research has shown that post-hemorrhagic hydrocephalus (PHH), another common cause, shares many similar pathophysiological pathways with PIH, namely through shared inflammatory mechanisms [125, 127, 128]. The build-up of CSF has been commonly attributed to intraventricular obstruction of both CSF flow and reabsorption (choroid plexitis, tumor formation, narrowing of passages such as the foramen of Monro, or ablation of arachnoid granulations) [128–130]. As such, the standard practice for hydrocephalus amelioration is through surgical methods such as placement of intraventricular shunts or endoscopic third ventriculostomy (ETV) to restore CSF flow. ETV may also be combined with choroid plexus cauterization to reduce CSF production. However, due to the invasive nature of treatment, complications often occur due to shunt failure or infections, and continued dependence on surgical intervention is required [131, 132]. Until recently, the role of CSF hypersecretion by the CP in the pathogenesis of hydrocephalus was minimally researched. Other secretory epithelial tissues have been shown to drastically increase the rate of fluid secretion when encountered by a pathogen or inflammatory environment, including intestinal and respiratory epithelial surfaces, suggesting the CP epithelium may respond in a similar manner [133, 134]. In 2017, Karimy et al. reported an inflammatory mechanism for the hypersecretion of CSF by the CP epithelium in PHH. The CSF hypersecretion observed in their rat model of PHH was mediated through the upregulation of the SPAK-NKCC1 co-transporter complex, which interestingly was dependent on the upstream signaling of the TLR4-NF-κB pathway [127]. This suggests that this mechanism of action may underlie CSF hypersecretion in PIH as TLR4 has been shown to activate under pathogenic conditions, specifically through the binding of LPS [62, 64, 122]. However, in contrast, the role of NKCC1 to mediate CSF clearance was shown in a mouse model of obstructive hydrocephalus and that overexpression of NKCC1 resulted in the reduction of ventriculomegaly [135]. The hypothesis of inflammatory induced CSF hypersecretion following infection and thus inducing PIH warrants further investigation as it may provide alternative, non-surgical methods for amelioration of hydrocephalus by pharmaceutical interventions such as TLR4 or NKCC1 inhibition.

### Zika virus

In adults, Zika virus (ZIKV) infection typically leads to mild symptoms including fever and joint pain; however, the ability of ZIKV to cross the placental barrier and infect the fetus has garnered considerable attention due to the severity of neurological injuries that can arise in the fetus. Early in development, the fetal brain is highly susceptible to infection once a pathogen has reached the CSF. This is because at early gestational periods, the ependymal lining of the ventricles have yet to fully form—in humans, ependymal differentiation occurs until 22 weeks of gestation, with complete maturation occurring postnatally [136, 137]. Prior to ependymal formation, neural progenitor cells (NPCs), which are found within the ventricular and subventricular zones, are in direct contact with the CSF. Several studies have shown that the ZIKV infects NPCs, inhibiting cellular differentiation and neurogenesis, and inducing cell death, leading to microcephaly [138–141]. While the impacts of ZIKV on neurodevelopment have been well studied, the route of entry into the CNS is less understood. Several lines of evidence have implicated the CP as a site of ZIKV trafficking from the blood to CSF. In in vivo non-human primate models, periventricular injury and damage to the ependymal lining is commonly found in congenital ZIKV infection [142–145]. Such injury patterns are also seen during human fetal neuroimaging and include ventriculomegaly and hypertrophy/cyst of the choroid plexus [146–148]. Although ZIKV infection is commonly associated with microcephaly, severe postnatal hydrocephalus can occur, suggesting that damage to the CSF and ventricular system may be involved [149]. However, there is no current understanding on how ZIKV impacts CSF production at a mechanistic level that provides insight into the pathogenesis of hydrocephalus. In a human cerebral organoid model system, the choroid plexus was found to be infected by the ZIKV [150]. A recent article by Kim et al. provided further evidence in CP involvement for ZIKV dissemination. In a mouse model for ZIKV brain infection, they observed that the ZIKV establishes a presence within the CP through infecting resident pericytes that adhere to the vasculature of the CP. This infection is soon followed by the emergence of the ZIKV within the CSF, and importantly precedes parenchymal infection [151]. In the same study, using an in vitro blood-CSF barrier model, they demonstrated that infected pericytes greatly enhanced the transmigration of ZIKV across the epithelial barrier and that secreted factors from the infected cells induced barrier disruption through a reduction in ZO-1 at cellular junctions [151]. The exact mechanism of entry for ZIKV to enter the CNS is still a work in progress (BBB vs BCSFB; direct transmigration vs “Trojan horse”), and as disease prognosis depends on key developmental milestones, additional research is needed to determine preferential dissemination pathways [152].

### John Cunningham virus

The John Cunningham virus (JCV, also known as Human polyomavirus 2), is widespread among the general population, with infection rates varying between 50 and 90% [153, 154]. The majority of infected individuals will show no clinical manifestations of infection as the virus tends to remain latent in secondary tissue sites such as the gastrointestinal tract or kidneys [154, 155]. In some cases, occasional shedding of JCV in the urine occurs [156–158]. However, JCV is the pathological agent responsible for progressive multifocal leukoencephalopathy (PML), an often fatal disease of the CNS that is characterized by progressive inflammation of the white matter, and through infection of oligodendrocytes and astrocytes, leads to demyelination [159, 160]. Although PML is rare, it almost exclusively occurs in the context of immunocompromised individuals, and as such up to 80% of PML patients occur in those with HIV [160, 161]. The progression of latent JCV to lytic invasion of the CNS and thus PML is poorly understood. It is suspected that at some point, reactivation of the latent virus within the secondary infected tissue leads to dissemination into the CNS, infecting glial cells responsible for inflammation and demyelination [154]. However, in some cases, latent JCV virus has been found to reside within the brain tissue of patients without PML [162]. Nevertheless, the route of dissemination into the CNS either during the lytic stage or prior to an established latent infection is not well understood. Although the blood–brain barrier has been suggested as a route of transmigration, and potentially through the infection of B-cells, the CP and the BCSFB have only recently been implicated in PML pathogenesis [163]. The JCV entry into cells has been shown to be dependent on two receptors, lactoseries tetrasaccharide c (LSTc) for initial cellular attachment, and a serotonin (5-HT)-2 receptor for cellular entry [164, 165]. Interestingly, distribution mapping of these receptors in both healthy and PML human samples indicated a lack of LSTc and thus no viral binding on oligodendrocytes and astrocytes; this is in contrast to both receptors and subsequently binding of the JCV occurring on kidney and CP epithelium tissue [166]. Furthermore, a case of fatal JCV meningitis with symptoms atypical of PML were characterized by communicating hydrocephalus and choroid plexus epithelial cells harboring productive JCV which is thought to be the cause of the high levels of viral load in the CSF [167]. These findings suggest viral infection of astrocytes and oligodendrocytes occurs independent of LSTc and that dissemination into the parenchyma may involve infection of CP epithelial cells. Indeed, the novel identification of extracellular vesicles derived by the CP epithelium has been shown to bridge CP involvement and LSTc-independent infection of parenchymal glia [87, 88]. Recent studies by O’Hara et al. highlight the susceptibility of CP epithelial cells to JCV infection and viral transmission via extracellular vesicles [88, 168]. In their investigation, they observed infected CP epithelial cells produced extracellular vesicles that contained JCV virions. Furthermore, it was shown that JCV is readily transmitted to glial cells through the uptake of these viral loaded vesicles in a receptor independent manner [88]. However, it is not well understood how JCV impacts CP epithelial cells following infection—studies aimed at barrier function and CP inflammatory relay to the brain are needed to attain a full picture of the effects of CP infection and CNS health.

### Human immunodeficiency virus

In 2021, it was estimated that 38 million people globally were living with human immunodeficiency virus (HIV; assume HIV-1 unless otherwise stated), with 1.5 million newly infected people reported that year [169]. HIV commonly infects host immune cells such as CD4 + T cells, macrophages, and dendritic cells through the binding of viral glycoproteins (gp160 and gp120) to host CD4 and chemokine receptors such as CCR5 or CXCR4 [170]. Due to the viral tropism towards host immune cells, HIV infection leads to critically low levels of CD4 + T cells, thus causing acquired immunodeficiency syndrome (AIDS) and increasing susceptibility to opportunistic infections. [171] Through the use of combination antiretroviral therapy (CART), suppression of HIV replication is possible, and many patients are capable of living with HIV without the progression towards AIDS. In fact, the development and administration of CART led to a substantial decrease in deaths while also halting the progression of HIV-associated neurocognitive disorder (HAND) in up to 77% of patients [172–176]. However, while some studies indicate beneficial outcomes of HAND following CART, the impact of the therapy on neurocognitive impairment is still poorly understood. Despite the most severe aspects of HAND [HIV-associated dementia (HAD)] being greatly reduced following the introduction of CART, the overall prevalence of HAND has not changed [177–182]. This is mainly caused by an increase in milder forms of HAND [asymptomatic neurocognitive impairment (ANI) and mild neurocognitive disorder (MND)] [177–182]. The prevalence of HAND can range from 20 to 50% and can occur in patients receiving CART, even when HIV RNA levels in the plasma are undetectable [177, 183, 184]. Although plasma levels of HIV RNA can be successfully controlled, other regions have been shown to act as reservoirs for HIV, including the genitourinary system, lymphoid tissue, and the CNS [185–188].

The phenomenon of HIV to escape into regions such as the CSF, termed CSF viral escape, has been observed to occur in 5–15% of CART patients [189–191]. While this may suggest a reservoir role for the brain, in recent years, the role of the choroid plexus has been largely ignored and needs to be revisited. Several studies in the 90’s and early 2000’s using post-mortem tissue found HIV infected immune cells situated within the stroma and supra-epithelial areas of the choroid plexus in approximately half of HIV cases [192–194]. These cells comprised of T lymphocytes, dendritic cells, and macrophages, and due to their apposition to capillaries, initial establishment of infection is thought to be of hematogenous origin [192–194]. Further evidence of the choroid plexus acting as a reservoir and key player in CNS pathogenesis comes from phylogenetic analysis of HIV in the brain, CP, and spleen of post-mortem tissue. In these studies, genotyping indicated that HIV from the brain and spleen formed distinct clusters based on the mutations of the HIV *env* and *pol* sequences, while HIV from the CP were found within each of the clusters, but with greater similarity towards brain sequences [195, 196]. Similarly, while spleen isolates displayed CCR5 and CXCR4 utilization, brain and CP isolates showed preferential utilization of CCR5, a major coreceptor for the infection of microglia [195–197]. In a more recent study, 44% of HIV-positive individuals showed BCSFB dysfunction, and similarly, individuals with CSF pleocytosis showed significant elevation in CSF inflammatory markers [198]. This suggests that the unique environment of the CP may provide selective pressure on mutations that confer drug resistance (mutations in *pol* sequence) and viral tropism towards the CNS through preferential utilization of host coreceptors (mutations in *env* sequence). Evolution of HIV within reservoir sites has seen on-going research, however, the CP has received little attention—such an important interface between the periphery and CNS requires greater focus in order to understand HIV evolution towards CNS tropism and drug resistance. While there has been research aimed at CNS penetrance of antiretroviral drugs and identification of transport systems at the CP, a greater focus on the CP would allow us to differentiate between the blood–CSF barrier and the BBB. This would provide insight into antiretroviral drug penetrance specifically across the blood-CSF barrier, allowing for more effective combinations of drugs that target the CP and CSF.

Research into feline immunodeficiency virus (FIV) and simian immunodeficiency virus (SIV) provides further contextual evidence that the CP may play a significant role in HIV neuropathology due to their similar mechanisms of infection [199, 200]. In an in vitro model system and in vivo, macrophages of the feline choroid plexus are infected by FIV, and effectively transfer the infection to T cells [201]. Furthermore, in a barrier model system of the feline CP, enhanced transmigration of macrophages and T cells are observed following FIV infection [202]. Similarly, in studies utilizing SIV in rhesus macaques, SIV is found within the CP, with an increased presence of macrophages and T cells within the stroma [203]. When rhesus macaques are infected with SIV of differing tropisms, lymphocyte- and macrophage-tropic viruses showed preferential infection of microglia [204].

### Borrelia burgdorferi

*Borrelia burgdorferi* (*Bb*), the etiological agent of Lyme disease, is estimated to infect up to 300,000 individuals in the US each year, with 30,000 cases being annually reported to the CDC [205–208]. *Bb* is transmitted to humans through the bite of a tick, and within a few days elicits symptoms similar to the flu, as well as the hallmark “bulls-eye” rash [209]. The bacteria invade secondary tissue through hematogenous dissemination, commonly residing in the extracellular matrix of joints – unilateral knee pain is typical in the manifestation of Lyme arthritis [209, 210]. Antibiotic treatment is highly effective in removing active infection, however, the efficacy can be time dependent. Following late or no treatment, persistent symptoms can occur even in the absence of infection [209, 211, 212]. This is noteworthy as it suggests that inflammatory mechanisms that were induced during infection persist–either through dysregulation in inflammatory pathways or continued induction through bacterial debris [213]. As *Bb* does not produce any known toxins, the inflammatory response is assumed to be the cause of tissue damage. Neurological manifestations, termed neuroborreliosis, are considered a late-stage symptom that leads to Bell’s palsy, lymphocytic meningitis, behavioral disorders (depression, fatigue, sleep disturbances), and overall cognitive decline [214–216]. *Bb* is not known to penetrate into the brain parenchyma; however, the bacteria can be found within the CSF of patients and colonize within the dura mater of mice [217–219]. It can be inferred that a likely mechanism of neurological manifestations occurs through the indirect induction of inflammation in the brain parenchyma. This may occur through invasion of peripheral immune cells or inflammatory cytokines released from the meninges or choroid plexus into the CSF that prompts inflammation within the brain. While the CNS pathology of neuroborreliosis is well-studied, it is unknown how *Bb* is able to enter the CNS. Traversal across the BBB has been studied as a potential route; however, there is limited evidence of parenchymal invasion. Recently, our lab sought to determine the effects of *Bb* on human CP epithelial cells in vitro [73]. Similar to other infections, we found that infection with *Bb* induced the production and secretion of inflammatory and chemotactic cytokines. Transcriptome analysis revealed reduced expression of barrier and scaffolding proteins, which may lead to a loss in barrier integrity. This suggests that infection with *Bb* and subsequent alterations to the BCSFB would promote an environment that allows for the migration of *Bb* and peripheral immune cells into the CSF. It is important to note such findings still need to be explored in vivo and the mechanism of CNS entry is yet to be determined. Furthermore, it is known that while *Bb* may not enter the brain parenchyma, the brain still undergoes inflammation during infection [220]. The mechanisms that underlie this indirect transmission of inflammatory signals is still unknown.

### SARS-CoV-2

Severe acute respiratory syndrome coronavirus 2 (SARS-CoV-2) is the virus that causes COVID-19, a respiratory illness that has led to the deaths of millions world-wide and is responsible for the on-going pandemic since late 2019. SARS-CoV-2 gains cellular entry through the binding of its spike protein S1 subunit to the ACE2 receptor which facilitates viral attachment to the cellular surface; cellular entry occurs through cleavage of the S1 subunit by the host cell protease TMPRSS2, exposing the S2 subunit that is needed for fusion [221, 222]. The virus predominantly infects epithelial tissue of the respiratory tract, but can be found to reside in other tissue including kidneys, intestines, and the brain [223, 224]. Recent studies have begun to examine and identify neurological complications in COVID-19 patients [225, 226]. Research into the prevalence of SARS-CoV-2 in the CNS of patients is ongoing, however, early studies suggest that viral presence in the CSF and brain parenchyma is a rare occurrence despite neurological symptoms being common [223–235]. Several possible routes of dissemination into the CNS have been suggested and studied, including transmigration across the olfactory mucosa, the enteric nervous system, the BBB, as well as the CP [236–240]. CP involvement in the pathogenesis of neurological manifestations of COVID-19 patients, with or without direct viral invasion of the CNS, is supported by several lines of evidence. Distribution mapping of the cellular components ACE2 and TMPRSS2 have shown a wide variety of expression in different organs, with expression levels within the brain being lower compared to other organs such as the lungs or small intestines [237, 241, 242]. Within the brain though, ACE2 was found to be expressed mainly on neurons, astrocytes, oligodendrocytes, and endothelial cells in distinct regions of the brain [243]. However, expression of ACE2 was notably higher within the choroid plexus of humans and mice [243]. Histological observations further substantiates the protein expression of ACE2 and TMPRSS2 on choroid plexus epithelial cells [237]. In human brain organoid models, SARS-CoV-2 shows neurotropic affinity to the choroid plexus epithelium with minimal or no infection of glia or neurons, and leads to disruption of the BCSFB [244, 245]. Furthermore, in a study of MS patients with COVID-19, researchers found SARS-CoV-2 (as well as ACE2) within CP epithelial cells and ependymal cells of both MS and non-MS patients with no evidence of neuronal and glial infection [246]. In contrast, single-cell transcriptome analysis of brain and CP samples from patients who had severe COVID-19 showed no molecular signs of SARS-CoV-2 [247]. However, results indicated robust expression in genes required for viral infection and substantial CP inflammation that is potentially relayed to the brain, resulting in inflammation [247]. These findings suggest that neurological complications from COVID-19 does not require the direct invasion of SARS-CoV-2 into the CNS and, in fact, it appears that neurological complications may more commonly arise as a result of aberrant inflammation throughout regions of the CNS, perhaps relayed by the CP. Findings in CSF samples from patients corroborate this hypothesis of a cytokine release syndrome, with rare observations of the virus but abnormal CSF findings that include increased CSF protein levels, elevated inflammatory factors (IL-8, IL-6, TNF-α), and CSF pleocytosis (neutrophilic and/or lymphocytic most commonly)—a systemic review of these findings was performed by Lewis et al. and Tandon et al. [227–229, 233, 248, 249]. As the pandemic continues, continued research into the neurological consequences of COVID-19 is needed in order to provide the necessary and potentially long-term care of patients.

## Conclusion—a target for therapeutics

Historically, therapeutics targeting the CNS have failed at much greater rates compared to non-CNS drugs and are further plagued by greater approval times and developmental times [250]. Nevertheless, the CP has received renewed interest for its therapeutic potential in part due to its unique anatomical position and its role as an immunological niche. As the CP is situated at the blood-CSF interface and is the main producer of CSF, designing vectors that target the CP epithelium would allow for the delivery of drugs to the CSF as well as providing a method to modulate its molecular composition [251]. This would allow for the bypass of the BBB and impact therapeutic targets that are located in regions such as the subarachnoid space and perivascular space, as well as the regions within the ventricular system. While this would lead to shallower penetration of drugs to the brain parenchyma, it would allow for broader penetration through acting upon the glia limitans. This would have the potential to control and ameliorate CNS inflammation [252, 253]. Although it is desirable to have a highly selective BCSFB, this presents a challenge in drug design as it may be difficult for the drug to cross into the CSF. The BBB and BCSFB contain transporters that actively efflux a range of drugs, one of which is P-glycoprotein I (P-gp)—inhibition of this transporter allows for the penetration of nelfinavir, an HIV antiviral, into the brain parenchyma [254, 255]. The importance of P-gp and similar transporters are not limited to drug penetration. In T cells, P-gp has been shown to be involved in the transmembrane transport of inflammatory and activating cytokines, IL-2, IL-4, and IFN-γ [256]. Following exposure to HIV pseudotype virus, T cells enhanced their expression of P-gp, leading to enhanced expression of TNFα, IFN-γ, and IL-6 [257]. Understanding the role of P-gp and other multidrug resistant proteins within immune cells and the CP would provide understanding to differential drug penetration and the relay of inflammatory mediators across the BCSFB. While studies have shown that the CP expresses several types of influx/efflux transporters necessary for the transport of metabolites, there have been minimal studies targeting these transporters to enhance the efficacy of drug delivery or regulation of inflammatory mediators [6, 258]. Additionally, as treatment options for hydrocephalus are limited to surgical interventions that focus on CSF flow obstruction, pharmaceutical inhibition of CSF production may prove efficacious in scenarios were surgical options fail or are not available.

During infection, the accumulation and activation of resident immune cells can promote deleterious inflammation leading to a compromise in barrier integrity. Modulation of the cytokine milieu, such as IFN-γ, has the potential to alter the immune cell population and inflammatory state during healthy and disease states [106, 127, 259]. Additionally, therapeutics targeting junctional proteins may protect against the breakdown of the BCSFB and thus prevent pathogen and immune cell invasion. Dexamethasone has been shown to prevent tight junction alterations during *S. suis* infection in vitro; however, its use in meningitis patients has seen mixed results [260, 261]. Nevertheless, a class of therapeutics that target the BCSFB would prove highly beneficial in maintaining CSF homeostasis.

The choroid plexus is a highly complex system whose cellular and molecular composition is still being unraveled. Understanding these complexities opens an entirely new route for therapeutic interventions. As the CSF surrounds the entirety of the CNS, it represents a critical environment that must be closely maintained. The CP and ventricular system provide a major avenue for CNS modulation in which future studies are needed to explore the many options of pharmaceutical targets and their downstream applications.

## Data Availability

Not applicable.

## Abbreviations

AIDS: Acquired immunodeficiency syndrome
BBB: Blood–brain barrier
BCSFB: Blood–cerebrospinal fluid barrier
*Bb*: *Borrelia burgdorferi*
CNS: Central nervous system
CSF: Cerebrospinal fluid
CP: Choroid plexus
CART: Combination antiretroviral therapy
DCs: Dendritic cells
ETV: Endoscopic third ventriculostomy
FIV: Feline immunodeficiency virus
HAND: HIV-associated neurocognitive disorder
HIV: Human immunodeficiency virus
JCV: John Cunningham virus
JAMs: Junctional adhesion molecules
LSTc: Lactoseries tetrasaccharide c
LPS: Lipopolysaccharide
MMPs: Matrix metalloproteases
NPCs: Neural progenitor cells
PAM: Pam_3_CSK_4_
PAMPS: Pathogen-associated molecular patterns
PRRs: Pattern recognition receptors
PHH: Post-hemorrhagic hydrocephalus
PIH: Post-infectious hydrocephalus
PML: Progressive multifocal leukoencephalopathy
SARS-CoV-2: Severe acute respiratory syndrome coronavirus 2
SIV: Simian immunodeficiency virus
TLRs: Toll-like receptors
